# Dynamics of Carbon and Soil Enzyme Activities under Arabica Coffee Intercropped with *Brachiaria decumbens* in the Brazilian Cerrado

**DOI:** 10.3390/plants13060835

**Published:** 2024-03-14

**Authors:** Thais Rodrigues de Sousa, Arminda Moreira de Carvalho, Maria Lucrécia Gerosa Ramos, Alexsandra Duarte de Oliveira, Douglas Rodrigues de Jesus, Ana Caroline Pereira da Fonseca, Fernanda Rodrigues da Costa Silva, Francisco Marcos dos Santos Delvico, Fábio Bueno dos Reis Junior, Robélio Leandro Marchão

**Affiliations:** 1 Faculty of Agronomy and Veterinary Medicine, University of Brasilia, Campus Darcy Ribeiro, Brasilia 70910-970, DF, Brazil; rodrigues-douglas@outlook.com (D.R.d.J.); caroline3fonseca@hotmail.com (A.C.P.d.F.); fernanda-srodrigues@hotmail.com (F.R.d.C.S.); 2 Embrapa Cerrados, BR-020, Km 18, Planaltina 73310-970, DF, Brazil; alexsandra.duarte@embrapa.br (A.D.d.O.); francisco.delvico@embrapa.br (F.M.d.S.D.); fabio.reis@embrapa.br (F.B.d.R.J.); robelio.marchao@embrapa.br (R.L.M.)

**Keywords:** land-use change, intercropping management system, soil organic carbon, soil quality

## Abstract

The change in land use in the Brazilian Cerrado modifies the dynamics of soil organic matter (SOM) and, consequently, carbon (C) stocks and their fractions and soil enzyme activities. This study evaluated the effect of brachiaria (*Brachiaria decumbens* Stapf.) intercropped with Arabica coffee (*Coffea arabica* L.) on the stock and fractions of soil carbon and enzyme activities. The experiment was arranged in a completely randomized block design with three replications and treatments in a factorial design. The first factor consisted of coffee with or without intercropped brachiaria, the second of Arabica coffee cultivars (‘I.P.R.103’ and ‘I.P.R.99’) and the third factor of the point of soil sampling (under the canopy (UC) and in inter-rows (I)). Soil was sampled in layers of 0–10, 10–20, 20–30, 30–40, 40–60 and 60–80 cm. Soil from the 0–10 cm layer was also used to analyze enzymatic activity. Significant effects of coffee intercropped with brachiaria were confirmed for particulate organic carbon (POC), with highest contents in the 0–10 and 20–30 cm layers (9.62 and 6.48 g kg^−1^, respectively), and for soil enzymes (280.83 and 180.3 μg p-nitrophenol g^−1^ for arylsulfatase and β-glucosidase, respectively).

## 1. Introduction

Coffee is one of the most important agricultural commodities in Brazil. If agricultural practices and management are adequate, the conversion of pasture areas into coffee plantations can eventually increase carbon (C) stocks and improve soil quality [1,2].

According to Mackay et al. [3], a 5% increase in soil C storage promotes a decrease in atmospheric carbon dioxide (CO_2_) of 16%, i.e., greenhouse gas (GHG) mitigation. Perennial species, as cultivated in agroforestry systems, are more efficient than annual crops to increase C and nitrogen (N) storage and mitigate global warming [4,5]. The mechanisms of soil C stabilization are associated with the physical protection of SOM, the formation of organomineral compounds and SOM maintenance [6].

Intercropping systems favor C accumulation in the soil profile [7,8,9] and make nutrient cycling more effective, mainly with regard to N [5], which improves the productivity of agricultural systems and soil quality [10]. When grasses such as brachiaria are planted, soil health is improved, nutrient use and cycling efficiency are intensified, soil protection is enhanced and the socio-economic contribution is increased [11]. Therefore, cover crops in agricultural systems represent a strategy for increasing SOM and improving the soil quality by storing C in the soil [12,13].

The biochemical composition of plant residues affects the dynamics of decomposition and nutrient cycling [14], microbial biomass formation [9] and soil enzymatic activity [15,16]. Soil management systems using cover crops can also alter the chemical and physical fractions of soil organic C [17]. The POC physical fraction consists mainly of decomposed relatively lighter plant material and is sensitive to soil management [17,18,19,20,21]. In addition to this management sensitivity, the humic fractions and chemical fractionation SOM represent a soil C reserve [22].

Soil management and cover crops can affect the soil enzyme activity, which consists of biological parameters. The activity of arylsulfatase increased significantly in soil under brachiaria intercropped with coffee [15]. Other factors other than soil management also control SOM content and quality, e.g., soil texture, vegetation cover, climate and particularly the biochemical composition and quantity of plant residues [12,23,24]. The biochemical composition of plant residues affects the dynamics of decomposition and nutrient cycling [24], microbial biomass formation [9] and enzymatic activity [15,16].

This study hypothesized that brachiaria (*Brachiaria decumbens*) intercropped with Arabica coffee in the Cerrado contributes to increasing the soil C stock, which improves the soil biochemical quality. Thus, this study evaluated the effect of brachiaria intercropped with Arabica coffee on the soil C stock, SOM fractions and enzyme activities in the Brazilian Cerrado.

## 2. Results and Discussion

### 2.1. Soil Carbon

Soil sampling was carried out under the coffee canopy and in between the rows for two coffee cultivars (‘I.P.R.103’ and ‘I.P.R.99’), but there was no effect of cultivars on soil carbon content. Due to the effects of fertilizer application on the coffee, the incidence of solar radiation in the inter-rows, irrigation and shading under the coffee canopy, soil samples were collected in and between the rows, as the contribution of root biomass from coffee cultivars and brachiaria can alter the soil properties. These factors influence crop residue decomposition and SOM mineralization and, consequently, the accumulation of soil C. There was no effect of cultivars on soil carbon content collected in and between the rows.

Rocha et al. [25] showed that under coffee–brachiaria intercropping compared with coffee monocultures, the soil physical properties under the coffee canopy differed, mainly with regard to aggregate stability, micropores and water storage.

### 2.2. Soil Total C Content

**Figure 1:**
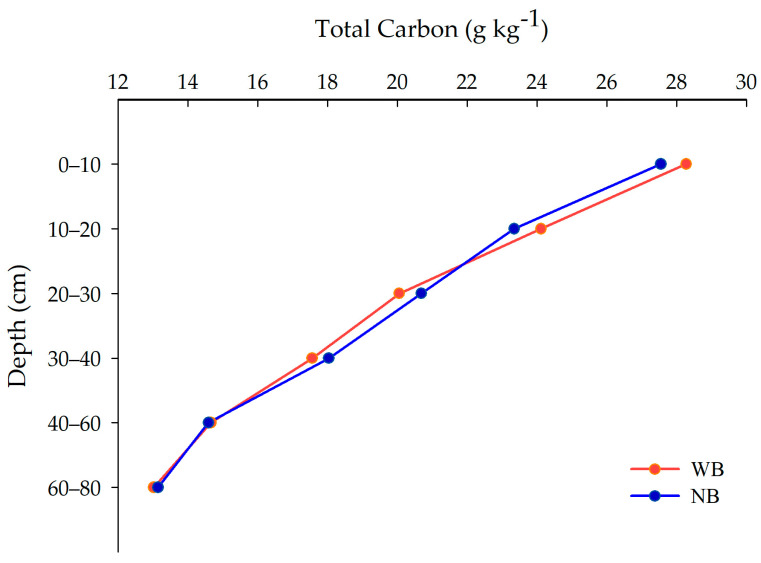
Total soil carbon (g kg^−1^) under Arabica coffee with and without intercropping with *Brachiaria decumbens* in the Cerrado. In red, the treatment WB (Arabica coffee intercropped with *Brachiaria decumbens*) and, in blue, the treatment NB (Arabica coffee without *Brachiaria decumbens*). Each point is the mean of two coffee cultivars (‘I.P.R.103’ and ‘I.P.R.99’).

Down to a depth of 80 cm, soil total carbon (TC) did not differ (*p* > 0.05) between treatments with and without brachiaria and, in relation to the sampling point, be it under the coffee canopy or in between the coffee rows, there was no effect of cultivars (‘I.P.R.103’ and ‘I.P.R.99’) on soil total carbon content (Figure 1). Brachiaria promoted an accumulation of TC of 28.28 and 24.12 g C kg^−1^, respectively, in the 0–10 and 10–20 cm layers (Figure 1), as similarly recorded in other studies [26,27], probably due to the root system of this grass. The highest TC value (28.28 g C kg^−1^) in the 0–10 cm layer (Figure 1) was possibly due to the higher deposition of organic residues in this surface layer [1]. In an evaluation of TC at a depth of 10 cm under irrigated coffee in an area contiguous with that of this study, Rocha et al. [2] found a higher value (29.12 g C kg^−1^) than in the 10–20 cm layer (23.44 g C kg^−1^).

In the coffee–brachiaria intercrop (CF-WB) system, TC contents were 28.28 and 13.03 g C kg^−1^, respectively, in the 0–10 cm and 60–80 cm layers, with similar values for the coffee monoculture without brachiaria (CF-NB) (27.55 and 13.15 g C kg^−1^, respectively) in the same layers (Figure 1).

### 2.3. Soil C Stock

Soil carbon stocks did not differ between treatments down to a depth of 80 cm. The C stock in the CF-WB treatment was 186.39 Mg ha^−1^ and without brachiaria was 184.17 Mg ha^−1^. These values were considered high because, in native Cerrado areas, C stocks down to a depth of 60 cm were between 120 and 219 Mg ha^−1^ [26]. In another study, in the state of Paraná in southern Brazil, where coffee was cultivated with rubber trees for 16 years, the C stock was 148.34 Mg ha^−1^ [1]. In the 0–60 cm layer of soils under no tillage with the presence of brachiaria (soybean/*Brachiaria ruziziensis*/soybean and soybean/*Brachiaria ruziziensis*), De Carvalho et al. [24] found higher soil C stocks than in native Cerrado, with gains of 5.2 and 4.4 Mg C ha^−1^, respectively. This can be explained by the high capacity of pastures for shoot production, the particularly well-developed root system [28] and the positive management effect when plant residues are left on the soil surface. Some brachiaria species grow well in the soils of the Brazilian Cerrado, producing large amounts of shoot and especially root biomass, and are efficient in storing C in the soil profile [28,29].

In this study, the presence and absence of brachiaria in coffee plantations was only implemented at the establishment of the coffee, which may explain the lack of a significant effect of presence or absence of brachiaria in between the coffee rows.

The quantity and quality of plant residues left on the soil may promote a positive effect on soil C stocks [14,21,24,30]. In addition, several edaphoclimatic, topographical and land-use change factors can alter C stocks and mitigate nitrous oxide (N_2_O) emissions, depending on the management system [24,31,32]. The presence of brachiaria intercropped with coffee in this study should increase the soil C stocks in the long term, as stated by Rocha et al. [2].

In general, the conversion of native areas to agricultural systems tends to decrease C stocks in the soil system, but agroforestry and organic production systems have great potential to increase C stocks in the soil [8,24,33,34].

### 2.4. Soil Organic Matter Fractions

The chemical and physical fractions of SOM were evaluated in the 0–10, 10–20 and 20–30 cm layers, and the treatments differed (*p* < 0.05) only in relation to the POC physical fraction (Table 1); there was no effect of cultivars (‘I.P.R.103’ and ‘I.P.R.99’) on soil carbon fractions. The POC contents were higher in coffee intercropped with *Brachiaria decumbens* in the 0–10 (9.62 g kg^−1^) and 20–30 cm (6.48 g kg^−1^) layers than in the coffee monoculture, with 7.38 g kg^−1^ and 5.10 g kg^−1^ in the 0–10 and 20–30 cm layers, respectively (Table 1). This result showed that POC was a C fraction that was more sensitive to changes in soil use and management [17,18,19,35] and that the presence of *Brachiaria decumbens* in intercrops with coffee promoted an increase in POC. In the surface 0–10 and the 20–30 cm layers, POC increased, possibly due to a greater contribution of *Brachiaria decumbens* residues on the surface and the effect of coffee and *Brachiaria decumbens* roots in the deeper layer.

In general, the two management systems with and without *Brachiaria decumbens* accumulated most C in the C-HUM chemical fraction. According to de Figueiredo et al. [36], these higher C levels in the HUM fraction may indicate greater chemical protection of SOM. Management systems of crops with pasture are more effective for increasing C stocks in the soil and mitigating N_2_O emissions [36,37].

### 2.5. Soil Enzymes

**Figure 2:**
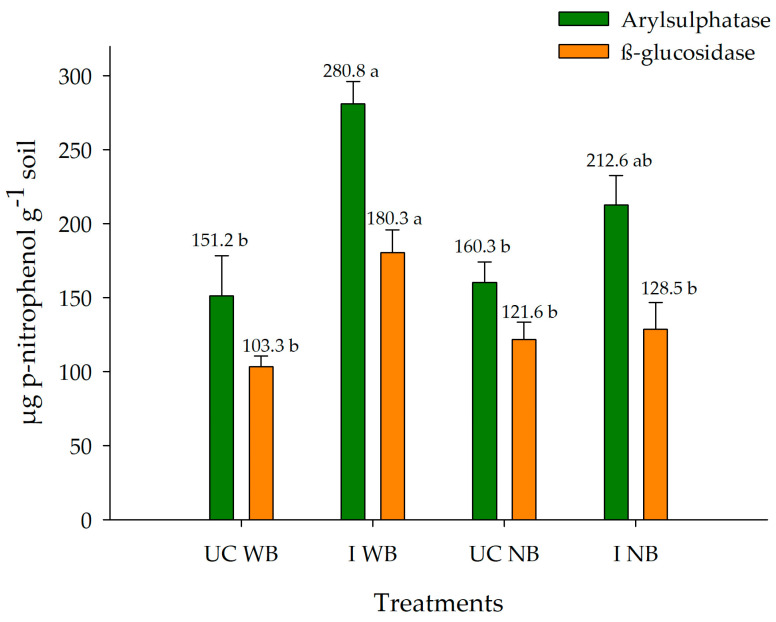
Enzymatic activity of β-glucosidase and arylsulfatase in soil under Arabica coffee with and without intercropping with *Brachiaria decumbens,* under the coffee tree canopy and in between the plant rows in the Cerrado, Planaltina-DF. UC WB—under the canopy of coffee trees intercropped with brachiaria; I WB—inter-rows between coffee intercropped with *Brachiaria decumbens*; UC NB—under the canopy of coffee cultivated without *Brachiaria decumbens;* I NB—coffee interrow without *Brachiaria decumbens* (data are means of soil collected of two coffee cultivars—‘I.P.R.103’ and ‘I.P.R.99’). Different letters indicate statistical differences between treatments by the Tukey’s test (*p* < 0.05).

There was no effect of coffee cultivars on the biological parameters of soil enzymes. In the coffee crops with and without *Brachiaria decumbens* in the inter-rows, where soil was sampled under the canopy and in the inter-rows, the enzymes arylsulfatase and β-glucosidase differed (*p* < 0.05) (Figure 2). The treatment with the highest enzymatic activity was obtained in the inter-rows of coffee intercropped with *Brachiaria decumbens* (IWB) (280.83 and 180.33 µg p-nitrophenol g^−1^ for arylsulfatase and β-glucosidase, respectively), followed by the inter-rows of coffee without *Brachiaria decumbens* (INB) (212.66 and 128.50 µg p-nitrophenol g^−1^ for arylsulfatase and β-glucosidase, respectively). However, these activities did not differ statistically from those observed in samples collected in the canopy projection. These values were higher than those reported by Rodrigues et al. and da Silva Aragão [15,16]. According to Rodrigues et al. [15], the activity of the soil enzymes arylsulfatase and β-glucosidase increased with the inclusion of *Brachiaria decumbens* in intercrops and in interaction with the water regime. Intercropping of *Brachiaria decumbens* with coffee increased the activity of arylsulfatase and β-glucosidase. This can be explained by the constant dropping of litter on the soil surface, resulting in more nutrients being incorporated in the soil. The higher activity of enzymes related to the presence of *Brachiaria decumbens* between the coffee rows can also be explained by the lower amplitude and fewer changes in soil temperature, in addition to the maintenance of moisture, which directly affects soil microorganisms [15].

To differentiate management systems by bioindicators, greater sensitivity of the enzymes arylsulfatase and β-glucosidase than of SOM was observed in 20 years of studies [35] and confirmed by the results of this study.

The results of this study suggest that arylsulfatase and β-glucosidase activities were favored by the plant biomass input in the coffee–brachiaria intercrop. In the five months of evaluation, the mean forage production was 3.90 t ha^−1^ of dry matter, an input that stimulated the microbial activity of SOM decomposition. Soil enzyme activities can raise coffee yields and are often related to the level of SOM content and quality [16]. This increase in enzymatic activity may also indicate the potential of the management under study to increase SOM [15].

Thus, the enzymes arylsulfatase and β-glucosidase are bioindicators capable of detecting changes in the biological functioning before significant changes become detectable in SOM contents or even part of C fractions, as shown in this study.

### 2.6. Principal Component Analysis

**Figure 3:**
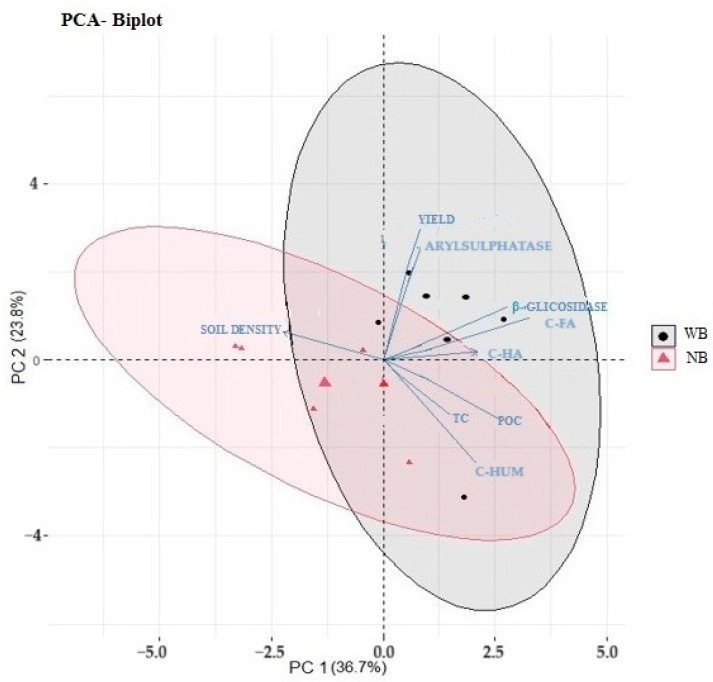
Analysis of principal components under management systems of Arabica coffee with and without intercrop with *Brachiaria decumbens* in the Cerrado.

Two principal components (PC1 and PC2) were generated as tools to distinguish the management systems (WB and NB) based on soil properties and yield (TC, POC, C-HA, C-FA, C-HUM, soil density, arylsulfatase, β-glucosidase and yield). The cumulative variation in the distribution of the selected variables was PC1 (36.7%) and PC2 (23.8%), i.e., a total of 60.5% for the sum of the principal components (Figure 3).

In the evaluated period, the first principal component (PC1) had the closest correlation with β-glucosidase (0.84), soil density (−0.77), C-HA (0.73), C-FA (0.70) and POC (0.62), while PC2 had the best correlation with TC (−0.584) (Table 2). The interaction analysis showed two groups of correlations: one with TC, POC and C-HUM (for PC1) and the other with the most variables such as soil enzymes, grain yield and SOM chemical fractions (for PC2) (Figure 3).

Arylsulfatase is strongly associated with coffee grain yield [16]. Principal component analysis suggested an integrated effect of the variables related to SOM and enzymatic activity, as shown by the clusters. In this context, three groups of correlations were observed. One group comprised yield and arylsulfatase, the second group β-glucosidase, C-FA and C-HA and a third group POC, TC and C-HUM.

In general, in relation to management systems, the systems with and without brachiaria were differentiated in this study. This separation shows that the management system without *Brachiaria decumbens* was more associated with organic C and the more humified C fraction and less subject to variations in soil management. On the other hand, the presence of *Brachiaria decumbens* in the intercropped system can improve soil properties and promote an accumulation of SOM fractions and indicators more sensitive to soil management changes (POC and enzymatic activity). This suggests differences between treatments with and without a cover crop (*Brachiaria decumbens*).

## 3. Materials and Methods

### 3.1. Experimental Location and Description

**Figure 4:**
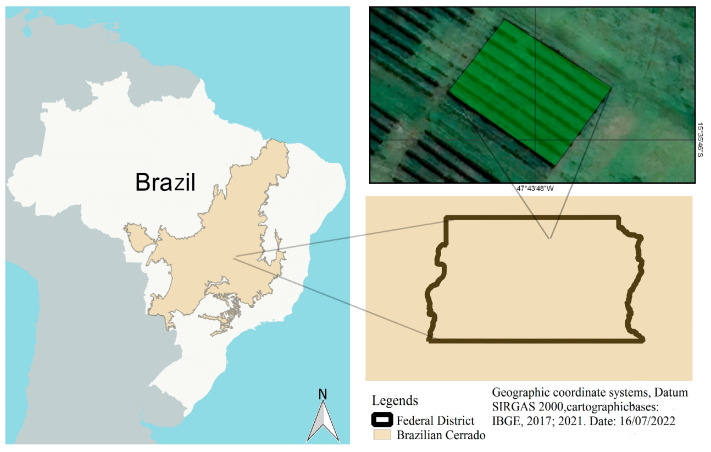
Location of the experimental area at Embrapa Cerrados in the Cerrado biome Planaltina, DF. Arabica coffee with and without the presence of *Brachiaria decumbens* in the coffee inter-rows.

The experiment was conducted in the experimental area of Embrapa Cerrados (UCAC), Planaltina-DF, Brazil (latitude 15°35′30″ S and longitude 47°42′30″ W) (Figure 4).

**Figure 5:**
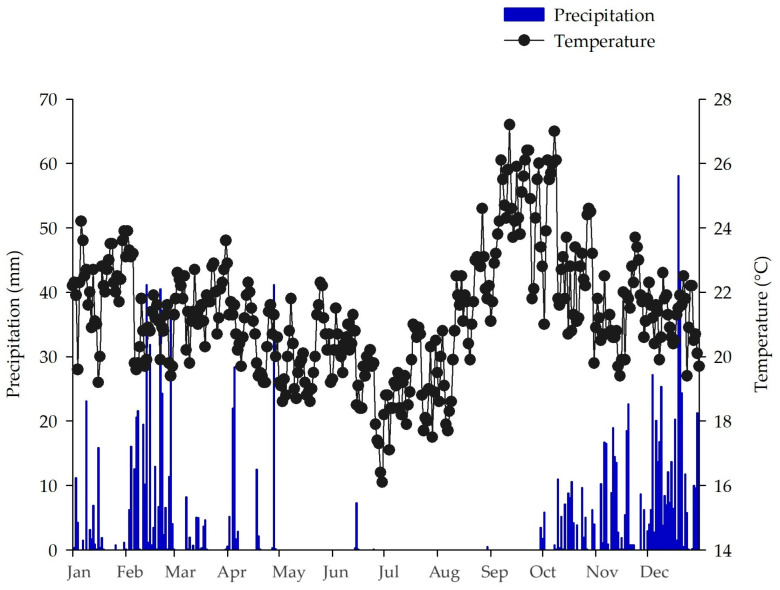
Average air temperature (°C) and average precipitation (mm) in the experimental area of Embrapa Cerrados from January to December 2021, in the administrative region of Planaltina, DF.

The regional climate is Aw, according to the Köppen classification [38], with two well-defined seasons (dry winters and humid summers). The average annual temperature varies from 22 to 25 °C and average precipitation is 1345.8 mm [39] (Figure 5) (80% of this precipitation occurs during the rainy season between October and February). The soil of the area is classified as Oxisol [40]. Before the experiment was installed, the soil chemical analysis (0–20 cm) determined the pH in water at a soil/solution ratio of 1:1 5.2; Al^3+^, Ca^2+^ and Mg^2+^ extracted by the KCl extracting solution was 1 mol L^−1^ 4.3, 22.9 and 8.3 mmolc dm^−3^, respectively; H + Al 76.0 mmolc dm^−3^; P and K extracted by the Mehlich-1 method 1.4 and 61.2 mg dm^−3^, respectively; Al saturation 12%. Particle size analysis detected mean levels of clay, silt and fine and coarse sand contents of 601, 116, 47 and 236 g kg^−1^, respectively.

From January 2000 to December 2007, brachiaria (synonymy of *Brachiaria decumbens*) was planted in the experimental area as a cover crop without grazing. Later, coffee cultivar Catuaí Vermelho 144 was planted and maintained until 2016. After harvest, the trees were renewed by drastic coffee tree pruning. In February 2019, two Arabica coffee cultivars (cvs. ‘I.P.R.103’ and ‘I.P.R.99’) were planted at a spacing of 3.5 m between rows and 0.5 m between plants. Two years after coffee planting, with and without *Brachiaria decumbens* between rows, the soil was sampled. Prior to this experiment, the previous coffee plantation was cultivated by intercropping with *Brachiaria decumbens.* Only at the time of implementation of this study, in February 2019, the treatments were separated into a coffee monoculture and a coffee–*Brachiaria decumbens* intercropping. Thus, soil was sampled two years after treatments with and without *Brachiaria decumbens* intercropping. In the inter-rows without *Brachiaria decumbens,* Indaziflan was applied with a backpack sprayer at a rate of 180 mL/ha^−1^ under the canopy, and weeds were removed manually. *Brachiaria decumbens* was planted with a wheat planter at a row spacing of 18 cm and at a seeding rate of 8 kg ha^−1^.

The experimental design was completely randomized with three replications, with treatments arranged in a factorial design. The first factor tested intercropping with (WB) and without (NB) *Brachiaria decumbens*, the second factor two Arabica coffee cultivars (‘I.P.R.103’ and ‘I.P.R.99’) and the third factor the sampling position (under the coffee canopy (UC) or in the coffee inter-rows (I)). Each plot consisted of eight plants.

The experiment was irrigated with a central pivot irrigation system and the management of irrigation was based on the soil water content and when the soil moisture corresponded to the consumption of 50% of the water available in the 0–10 cm layer [41]. Between May and early September (dry season), water stress was applied for 60 days to induce uniform flowering after resuming irrigation [42]. Water content was monitored with ML1 (Delta-T Devices) moisture probes.

The plots with *Brachiaria decumbens* intercropped between the coffee rows were cut when the forage height reached 0.60 m and the crop residues were left on the soil surface. The following treatments were evaluated: UCWB-‘IPR103’ (under the canopy of coffee cv. IPR-103 intercropped with *Brachiaria decumbens*); UCWB-‘IPR99’ (under the canopy of coffee cv. IPR-99 intercropped with brachiaria); UCNB-‘IPR103’ (under the canopy of coffee cv. IPR-103, without brachiaria); UCNB-‘IPR99’ (under the canopy of coffee cv. IPR-99, without brachiaria) IWB-‘IPR103’ (inter-rows of coffee cv. IPR-103, intercropped with *Brachiaria decumbens*); IWB-‘IPR99’ (inter-rows of coffee cv. IPR-99, intercropped with *Brachiaria decumbens*); INB-‘IPR103’ (inter-rows of coffee cv. IPR-103, without brachiaria) (inter-rows of coffee cv. IPR-99, without brachiaria).

Fertilization at coffee planting consisted of 300 kg ha^−1^ of triple superphosphate (41% of P_2_O_5_). In the first year, sidedressing consisted of 200 kg N ha^−1^ in urea and 200 kg K_2_O ha^−1^ in potassium chloride, applied in September, November, January and March. In the second year, 200 kg N ha^−1^ in urea and 200 kg K_2_O ha^−1^ in potassium chloride was sidedressed four times (January, March, September and November), plus 50 kg ha^−1^ of fritted trace elements (FTE-BR 12). From the third year onwards, double rates of the initial sidedressing were applied (400 kg N ha^−1^, 400 kg K_2_O ha^−1^ and 100 kg ha^−1^ of FTE-BR 12, applied four times (in September, November, January and March) for N and K). Phosphorus was sidedressed in the coffee rows, i.e., 2/3 in September when irrigation was resumed and 1/3 between November and December. Liming consisted of 2 t ha^−1^ of dolomitic limestone and 2 t ha^−1^ agricultural gypsum after harvest in 2021.

### 3.2. Soil Collection and Analysis

**Figure 6:**
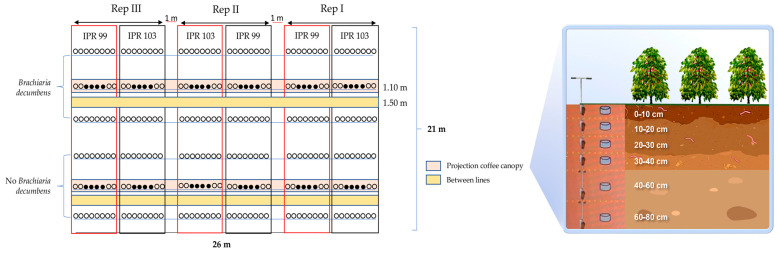
Sketch of the experimental area. Yellow, soil sampling in the coffee inter-rows. Pink, sampling under the coffee canopy. The black circles highlighted with the pink band correspond to the plot’s useful area, while the white circles correspond to the plot’s border. The plots highlighted were used in this experiment to collect soil samples (pink is the canopy projection, and yellow is the interrow of the coffee tree.

Soil was sampled under the coffee canopy and between the coffee rows of the two Arabica cultivars (cvs. ‘I.P.R.103’ and ‘I.P.R.99’) from the 0–10, 10–20, 20–30, 30–40, 40–60 and 60–80 cm layers with a Dutch auger (Figure 6). Samples were collected on the first and second of December 2021, two years after coffee pruning and implementation of the system with and without *Brachiaria decumbens*. Each sample consisted of six subsamples from each replication.

From the coffee rows, six subsamples per replication were taken from the central region of the rows. Of the soil sampled from under the coffee canopy, six subsamples were blended into a composite sample to represent the strip where fertilizers were applied. Then, the samples were crushed and smoothed, stored in plastic bags and transported to the laboratory of Embrapa Cerrados in a hermetically sealed ice box for further analysis. Part of the soil samples from the 0–10 cm layer was reserved for analysis of enzyme activities (β-glucosidase and arylsulfatase).

To determine bulk density, two trenches were dug in the coffee inter-rows of the experimental area with (WB) and without (NB) *Brachiaria decumbens.* Undisturbed soil samples were collected with volumetric rings from the 0–10, 10–20, 20–30, 30–40, 40–60 and 60–80 cm layers, with four replications (two in the middle and two on either side of the trench). The material was oven-dried at 105 °C. The soil density was calculated by measuring the internal volume of the cylinder, as expressed in Equation (1).

(1) $$Ds=\frac{Ma}{V}$$

where

*Ds* is soil density in g cm^−3^; *Ma* is weight of soil sample dried at 105 °C to constant weight in g; *V* is cylinder volume in cm^3^.

### 3.3. Soil C Analysis

The soil samples were air-dried and sieved (<2 mm). To determine total C (TC), the samples were ground with pestle and mortar, and the soil was sieved (<150 µm).

The physical particle–size fractionation was performed according to Cambardella et al. [43]. A total of 50 g of sieved soil samples (<2 mm) were added to 500 mL flasks with 175 mL of a sodium hexametaphosphate solution (5 g L^−1^). The suspension was stirred for 15 h at 130 rpm, passed through a 53 µm mesh sieve and rinsed with tap water. The soil retained in the sieve (>53 µm) was dried in an oven at 45 °C and weighed until constant weight. The soil was ground with pestle and mortar and sieved (<150 µm), and carbon content in this fraction (POC) was determined.

Total carbon and POC were analyzed by dry combustion in an elemental analyzer model 2400 Series II CHNS at the Laboratory of Soils of Embrapa Cerrados by high-temperature oxidation (1000 °C).

For the determination of C stock, C concentrations were converted into C stock for each sampled layer, according to Ferreira et al. [44] (Equation (2)).

Carbon Stock = (TC × BD × d)/10(2)

where TC is soil total carbon (g kg^−1^), BD is bulk density (g cm^−3^) and d is the soil layer (cm).

#### 3.3.1. Humic Substances

For the chemical fractionation of SOM, the differential solubility procedure proposed by Swift [45] was used. The solution was stirred for 4 h at 80 rpm and, after 12 h, was centrifuged at 4000 rpm for 30 min. The supernatant was collected and reserved in a separate flask. A further 20 mL of 0.5 mol L^−1^ NaOH was added to the soil retained in the same centrifuge tubes. The material was stirred for 2.5 h at 80 rpm and centrifuged at 4000 rpm for 30 min. The supernatant was added to the reserved one, thus forming an alkaline extract. The fulvic acid fraction was soluble in the alkaline extract, and the humic acid fraction corresponded to the precipitated portion after decreasing the extract to a pH between 1 and 1.5. The humin fraction was considered as all the residue insoluble in acid and alkaline medium retained in the centrifuge tube after centrifugation of the alkaline extractant. The C contents in the extracts of the fulvic acid (C-FA), humic acid (C-HA) and humin (C-HUM) fractions were determined as described by Yeomans and Bremner [46], with modifications, where the samples were heated to 150 °C in block test tubes, by C oxidation with potassium dichromate (K_2_Cr_2_O_7_) 0.042 mol L^−1^ for C-FA and C-HA and 0.167 mol L^−1^ for C-HUM and titrated with ferrous ammonium sulfate (Fe(NH_4_)_2_(SO_4_)_26_H_2_O). Based on this fractionation, C-FA, C-HA and C-HUM were calculated.

#### 3.3.2. Enzymatic Activity

The arylsulfatase and β-glucosidase activities were determined at the Soil Microbiology Laboratory of Embrapa Cerrados. Soil samples were air-dried at room temperature and sieved (<2 mm). The arylsulfatase and β-glucosidase activities were tested according to the methodology of Tabaitabai [47], a colorimetry method of p-nitrofenol determination released after soil incubation at 37 °C for one hour in a specific substrate (buffered solution of p-nitrophenyl-β-D-glucopyranoside for β-glucosidase and buffered solution of p-nitrophenyl sulfate for arylsulfatase) The samples were read in a spectrophotometer (UV-1800 Shimadzu, UV-1800, Kyoto, Japan) at 420 nm for β-glucosidase and 410 nm for arylsulfatase. The results were expressed in µg p-nitrophenol g^−1^ soil (mg PNP kg^−1^ h^−1^).

### 3.4. Statistical Analysis

The effect of management with coffee monoculture or coffee-*Brachiaria decumbens* intercropping, with two coffee cultivars (‘I.P.R.103’ and ‘I.P.R.99’), on soil properties were subjected to a Shapiro–Wilk normality test. In the analysis of variance (ANOVA), the sources of variation were plot with and without *Brachiaria decumbens* in the inter-rows, subplot Arabica coffee cultivars (cvs. ‘I.P.R.103’ and ‘I.P.R.99’) and the sub-subplot under the canopy and coffee inter-row.

Principal component analysis (PCA) was performed to evaluate the possible relationships between soil properties and management in the study area.

Analysis of variance and PCA were performed in the statistical program R version 3.5.0 [48] and the means were compared by Tukey’s test.

## 4. Conclusions

The results show an integrated effect of variables related to MOS and enzymatic activity, with three groups being observed, represented by yield and arylsulfatase, β-glucosidase, C-FA and C-HA and a third group POC, TC and C-HUM. The results indicate an improvement in the biochemical quality of the soil due to the presence of *Brachiaria decumbens* in consortium with coffee, contributing to medium-to-long term low C coffee farming and its sustainability in the Cerrado.

## Figures and Tables

**Table 1:** Carbon fractions in different soil layers (0–10, 10–20 and 20–30 cm) under coffee intercropped with *Brachiaria decumbens* or coffee monoculture in the Cerrado biome. Particulate organic carbon (POC); fulvic acid carbon (C-FA); humic acid carbon (C-HA); and humin carbon (C-HUM) in g kg^−1^.

Layer	POC		C-FA		C-HA		C-HUM	
	WB	NB	WB	NB	WB	NB	WB	NB
0–10	9.62 a	7.38 b	0.12	0.12	0.08	0.10	3.73	3.72
CV (%)	23.59		6.74		10.28		2.82	
10–20	7.85	6.50	0.10	0.10	0.11	0.08	3.67	3.63
CV (%)	26.7		8.79		25.63		3.68	
20–30	6.48 a	5.10 b	0.08	0.08	0.05	0.05	3.17	3.57
CV (%)	19.96		7.63		38.02		20.68	

Different letters indicate statistical differences between treatments by the Tukey’s test (*p* < 0.05). The data represent means of soil under rows and inter-rows of the coffee cultivars ‘I.P.R.103’ and ‘I.P.R.99’. WB (Arabica coffee intercropped with *Brachiaria decumbens*) and NB (Arabica coffee *without Brachiaria decumbens*).

**Table 2:** Correlation coefficients between variables and principal component analysis (PC1 and PC2) for different managements in coffee.

Statistics	PC1	PC2
Eigen Value	3.3	2.1
% Explained	36.7	23.8
**Variables**	**PC1**	**PC2**
POC	0.62	−0.280
C-FA	0.70	0.158
C-HA	0.73	0.066
C-HUM	0.36	−0.392
TC	0.59	−0.584
ARYLSULPHATASE	0.26	0.852
β-GLUCOSIDASE	0.84	0.332
SOIL DENSITY	−0.77	0.222
YIELD	0.20	0.806

## Data Availability

The data presented in this study are available on request from the corresponding author.
